# SUMOylation of the Hyperpolarization-Activated Cyclic Nucleotide-Gated Channel 2 Increases Surface Expression and the Maximal Conductance of the Hyperpolarization-Activated Current

**DOI:** 10.3389/fnmol.2016.00168

**Published:** 2017-01-12

**Authors:** Anna R. Parker, Meghyn A. Welch, Lori A. Forster, Sarah M. Tasneem, Janhavi A. Dubhashi, Deborah J. Baro

**Affiliations:** ^1^Department of Biology, Georgia State UniversityAtlanta, GA, USA; ^2^Neuroscience Institute, Georgia State UniversityAtlanta, GA, USA

**Keywords:** SUMO, HCN, trafficking, ion channel, hyperpolarization-activated cyclic nucleotide-gated channel, cyclic nucleotide binding domain

## Abstract

Small Ubiquitin-like Modifier (SUMO) is a ∼10 kDa peptide that can be post-translationally added to a lysine (K) on a target protein to facilitate protein–protein interactions. Recent studies have found that SUMOylation can be regulated in an activity-dependent manner and that ion channel SUMOylation can alter the biophysical properties and surface expression of the channel. Hyperpolarization-activated cyclic nucleotide-gated (HCN) channel surface expression can be regulated in an activity-dependent manner through unknown processes. We hypothesized that SUMOylation might influence the surface expression of HCN2 channels. In this manuscript, we show that HCN2 channels are SUMOylated in the mouse brain. Baseline levels of SUMOylation were also observed for a GFP-tagged HCN2 channel stably expressed in Human embryonic kidney (Hek) cells. Elevating GFP-HCN2 channel SUMOylation above baseline in Hek cells led to an increase in surface expression that augmented the hyperpolarization-activated current (*I*_h_) mediated by these channels. Increased SUMOylation did not alter *I*_h_ voltage-dependence or kinetics of activation. There are five predicted intracellular SUMOylation sites on HCN2. Site-directed mutagenesis indicated that more than one K on the GFP-HCN2 channel was SUMOylated. Enhancing SUMOylation at one of the five predicted sites, K669, led to the increase in surface expression and *I*_h_ *G*_max_. The role of SUMOylation at additional sites is currently unknown. The SUMOylation site at K669 is also conserved in HCN1 channels. Aberrant SUMOylation has been linked to neurological diseases that also display alterations in HCN1 and HCN2 channel expression, such as seizures and Parkinson’s disease. This work is the first report that HCN channels can be SUMOylated and that this can regulate surface expression and *I*_h_.

## Introduction

Post-translational modifications can rapidly regulate proteins. SUMOylation is one such modification that is essential for most organisms (Flotho and Melchior, 2013). Small Ubiquitin-like Modifier (SUMO, a.k.a. Sentrin) is a ∼100 amino acid peptide that is covalently added to a lysine (K) residue on a target protein. The addition involves several steps. First, the immature SUMO protein is cleaved by a sentrin specific protease (SENP), exposing a C-terminal diglycine (Mukhopadhyay and Dasso, 2007). The SUMO E1 activating enzyme then transfers the mature SUMO peptide to the E2 conjugating enzyme, Ubc9, which will then add SUMO to the target protein (Desterro et al., 1997). SUMO can also be deconjugated from a target protein by SENP (Hickey et al., 2012). In mammals, SUMO is encoded by a family of four genes, termed SUMO1–4 (Flotho and Melchior, 2013). SUMO2 and SUMO3 proteins are ∼97% identical and are not readily distinguishable in most experiments. SUMO1 and SUMO2/3 proteins share ∼46% identity, and their sets of targets greatly overlap. SUMO4 cannot be cleaved into the mature form by SENP, and its function is unclear.

Modification by SUMO is subject to numerous forms of regulation. The availability of the SUMOylation site on a target protein can be regulated by the presence of other post-translational modifications. For example, phosphorylation near the SUMOylation site can either inhibit or enhance SUMOylation (Bossis and Melchior, 2006; Konopacki et al., 2011). SUMOylation can also be regulated by the availability of SUMO and/or SUMOylation enzymes (Loriol et al., 2013).

SUMOylation mediates protein–protein interactions (Makhnevych et al., 2009; Flotho and Melchior, 2013). As such, SUMO can coordinately regulate many diverse cellular processes ranging from DNA repair in the nucleus to signal transduction at the plasma membrane (Hickey et al., 2012). The effects of SUMO are observed throughout the neuron (Henley et al., 2014). SUMOylation can influence neuronal transcription by controlling the stability of transcription factors; for example, BMAL1 ubiquitination and degradation is enhanced when it is SUMOylated (Lee et al., 2008). SUMO can also regulate synaptic release. SUMOylation of the synaptic vesicle protein, RIM1α, promotes its direct interaction with Ca_v_2.1 channels, causing them to form clusters, which leads to rapid exocytosis of synaptic vesicles (Girach et al., 2013). In addition, SUMOylation plays a role in the trafficking of integral membrane proteins such as kainate receptors. SUMOylation of GluK2 is required for agonist-induced internalization of the kainate receptor, likely by facilitating its interaction with scaffolding and/or trafficking proteins (Konopacki et al., 2011; Chamberlain et al., 2012). However, the distinct interaction that leads to GluK2 internalization is still unknown. Voltage-gated ion channels are fundamental constituents of the neuronal membrane. Their trafficking is complex and highly regulated. The functions of SUMOylation in voltage-gated ion channel trafficking are largely unknown. We are interested in the role of SUMOylation in the regulated trafficking of hyperpolarization-activated cyclic nucleotide gated (HCN) ion channels.

HCN channels play a pivotal role in shaping neuronal excitability and synaptic integration by influencing several neuronal activity features including membrane potential, firing threshold, resonance frequency, temporal summation, and synaptic strength (Hutcheon and Yarom, 2000; Wahl-Schott and Biel, 2009; Shah, 2014). The mammalian HCN1–4 gene family encodes distinct channel isoforms (He et al., 2014). All isoforms are permeable to K^+^ and Na^+^, activate upon hyperpolarization, and mediate a slowly depolarizing current termed the hyperpolarization-activated current (*I*_h_). Besides being activated at hyperpolarized potentials, HCN channels can also be gated by the binding of cyclic nucleotides to the C-terminal cyclic nucleotide binding domain (CNBD) found in all isoforms (Robinson and Siegelbaum, 2003; He et al., 2014).

HCN channel isoforms differ in their biophysical properties and modulation by cAMP. Under basal conditions, the CNBD inhibits hyperpolarization-gating to a different extent in each isoform due to its isoform-specific interactions with the core transmembrane domain and the C-linker that connects the CNBD to the transmembrane domain (Wang et al., 2001). This variable inhibition results in isoform-specific steady-state activation curves. For example, the steady-state activation curve of HCN2 channels is 20 mV more hyperpolarized compared with HCN1 channels. Binding of cAMP to the CNBD relieves its inhibition on hyperpolarization-gating. Because of the isoform-specific interactions between the CNBD and the C-linker, the effect of cAMP binding will vary with the isoform (Wang et al., 2001). For example, binding of cAMP to the CNBD of HCN2 and HCN1 channels shifts their respective activation curves to more positive potentials, but HCN2 channels display a 17 mV shift while HCN1 channels display a 4 mV shift. Maximal effects of cAMP binding are observed for HCN2 and HCN4 isoforms. The activation kinetics also varies between isoforms with HCN1 and HCN4 having the fastest and slowest activation kinetics, respectively.

In addition to their distinct biophysical properties and cAMP modulation, each of the four isoforms also displays a unique expression pattern in the nervous system (He et al., 2014). HCN1 is highly enriched in the neocortex, hippocampus, cerebellar cortex, and brainstem. HCN2 is widely expressed in most brain regions, while HCN3 has low expression levels in the nervous system. HCN4 expression mirrors HCN1 and is also selectively expressed in several thalamic nuclei and neuronal populations in the basal ganglia and habenular cortex. The formation of heteromeric channels further enhances the complexity of *I*_h_ function and modulation *in vivo*.

HCN channel surface expression throughout the nervous system can be adjusted over several time courses (Zha et al., 2008; Shah, 2014; Furst and D’Avanzo, 2015; Smith et al., 2015; Brennan et al., 2016). Regulated SUMOylation could play a role in the trafficking of HCN channels. Since the trafficking of HCN2 channels has been fairly well studied (see Discussion), here we examine SUMOylation of HCN2 channels.

## Materials and Methods

### Drugs

All drugs were obtained from Sigma-Aldrich with the exception of Tween 20 (Fisher Scientific).

### Mouse Brain Membrane Preparations

#### Non-denaturing

A single mouse forebrain was homogenized on ice in homogenization buffer (5 mM NaH_2_PO_4_ buffer, 0.32 M sucrose) supplemented with protease inhibitor cocktail (1:100, Sigma cat. #P8340) and 20 mM *N*-Ethylmaleimide (NEM) to prevent SUMO deconjugation (Suzuki et al., 1999). Cell debris was pelleted at 5,000 rpm for 10 min at 4°C, and the supernatant was retained and further centrifuged at 40,000 rpm for 90 min at 4°C to pellet membrane-bound proteins. The supernatant was removed and the pellet containing intracellular and extracellular membrane bound proteins was resuspended in 1 ml of resuspension buffer (0.5% SDS, 5 nM NaH_2_PO_4_, protease inhibitor cocktail at 1:100) followed by shaking at 4°C for 1 h.

#### Denaturing

Tissue homogenization and centrifugation were the same as for the non-denaturing preparation. The membrane pellet was resuspended in 100 μl of denaturing buffer (2% SDS, 50 mM Tris-HCl pH7.5, 5 mM DTT) followed by shaking at 4°C for 1 h. The preparation was then diluted to 1 ml total volume with dH_2_O and boiled for 10 min. In all cases, protein concentration was determined with a bicinchoninic acid assay (BCA Assay, Pierce BCA Protein Assay Kit). Mouse brain membrane fractions were obtained from whole mouse forebrain tissue generously provided by Dr. Chun Jiang. All animal procedures were conducted in compliance with the regulation of the Institutional Animal Care and Use Committee of Georgia State University.

### Plasmids and Antibodies

A previously described mouse GFP-HCN2 fusion plasmid (Santoro et al., 2004) was generously provided by Dr. Bina Santoro’s Lab. Plasmids for transient transfection include the following: mCherry2-C1 was a gift from Michael Davidson (Addgene plasmid # 54563), Ubc9 (Yasugi and Howley, 1996) was a gift from Peter Howley (Addgene plasmid # 14438), SENP1 (Cheng et al., 2007) was a gift from Edward Yeh (Addgene plasmid # 17357), and SUMO2 (Kamitani et al., 1998) was a gift from Edward Yeh (Addgene plasmid # 17360). Antibodies used are shown in **Table 1**, and the specificity of each antibody was verified as indicated in the table.

**Table 1:** Primary antibodies.

Antigen	Verification of specificity	Manufacturer, species, cat no.	Concentration used
**SUMO2/3**	Specificity verified by company through Western blot (WB) analysis of Hek-293 cells transfected with SUMO2	Santa Cruz Biotechnology, rabbit polyclonal, cat. no. sc-32873	WB: 1:3000
SUMO1	Specificity verified by company through WB analysis of Hek-293 with and without SUMO1 expressed	Santa Cruz Biotechnology, rabbit polyclonal, sc-9060	WB: 1:3000
GFP (for WB)	Specificity verified by company through WB analysis of cells with and without GFP expressed	Santa Cruz Biotechnology, rabbit polyclonal, cat. no. sc-8334	WB: 1:4000
GFP (for IP)	We verified specificity through WB analysis (**Figure 2**)	Abcam, rabbit polyclonal, cat. no. ab290	1 μl per 500 μg of lysate
HCN2	Specificity verified by company through WB analysis of cells with and without HCN2 expressed	Santa Cruz Biotechnology, goat polyclonal, sc-19708	WB: 1:3000 (Hek cells)
HCN2	Specificity verified by company through WB analysis of mouse and rat membrane proteins	UC Davis/NIH NeuroMab, mouse monoclonal, N71/37	WB 1:200 (Mouse brain)
Actin	Specificity verified by company through WB analysis of non-transfected and β-Actin transfected Hek-293 cells	Santa Cruz Biotechnology, rabbit polyclonal, sc-1616-R	WB: 1:2000
Na^+^/K^+^-ATPase	Specificity verified by company through WB analysis of Human, Mouse and Rat tissues expressing the Na^+^/K^+^-ATPase	Abcam, mouse monoclonal, cat. no. ab7671	WB: 1:3000

### Site-Directed Mutagenesis

PCR was used to create two site-directed mutations in the GFP-HCN2 fusion plasmid described above: K534R and K669R. The two sets of primers are listed in **Table 2**. PrimeStar GXL polymerase (Takara) was used along with the buffer, nucleotides, and instructions supplied by the manufacturer. Typically, 10 ng of plasmid DNA served as the template in a 50 μl reaction. The cycling conditions were: 1x 98°C, 1 min; 30x 98°C, 30 s, 68°C, 7 min; 1x 68°C, 5 min. Upon completion, 20 units of DpnI (Clontech) were added to the reaction, which was incubated at 37°C for 1–2 h to digest the template DNA. Afterward, 1–2 μl of the reaction was added to a 50 μl aliquot of subcloning grade competent XL1blue cells (Agilent) and incubated on ice for 30 min. The cells were then heat-shocked at 42°C for 45 s. NZY broth (100 μl) was added, and the cells were incubated at 37°C for 20 min to allow for expression of kanamycin resistance. Cells were then plated on NZY plates containing 30 μg/ml kanamycin and incubated at 37°C overnight. Plasmid DNA from resultant colonies was isolated (Qiagen), the insert was sequenced in its entirety, and analyzed with Lasergene software (DNAstar) to ensure that only the intended mutation was generated. All sequencing was done by the Georgia State University Cell Protein and DNA Core Facilities. To create multiple mutations, a previously mutated GFP-HCN2 fusion plasmid was used as the template for a PCR with a different primer.

**Table 2:** Site directed mutagenesis primers.

Primer name	Primer sequence
mHCN2K534RFor	5′-CACAGCCATGCTGACAAAGCTCAg ATTTGAGGTCTTCCAGCCTGG-3’
mHCN2K534RRev	5′-CCAGGCTGGAAGACCTCAAATcTGAGCTTT GTCAGCATGGCTGTG-3’
mHCN2K669RFor	5′-GCCATCATCCAGGAGATTGTCAgATATGACCG TGAGATGGTGCAGC-3’
mHCN2K669RRev	5′-GCTGCACCATCTCACGGTCATATcTGACA ATCTCCTGGATGATGGC-3’

### Cell Culture, Stable, and Transient Transfections

Hek-293 cells and all cell culture reagents were obtained from American Type Culture Collection (ATCC; Manassas, VA, USA). Hek-293 cells were cultured at 37°C, and 5% CO_2_ in EMEM media supplemented with 10% fetal bovine serum and 1% Penicillin-Streptomycin.

#### Transfections to Produce Stable Lines

A cell line stably expressing a GFP-HCN2 fusion protein (Hek-HCN2) was generated by transfecting a 60 mm plate of Hek-293 cells with the GFP-HCN2 plasmid using Lipofectamine 2000 (Invitrogen) according to instructions provided by the manufacturer. After 2 days, transfected cells were trypsinized, resuspended, and replated to 60 mm plates (5–20 μl of a 1 ml cell resuspension per 60 mm plate) and the selection agent, G418 (Geneticin, Gibco, 500 μg/ml), was added to the media. After 3–5 weeks, individual colonies were selected using cloning rings. Stable expression was checked by fluorescence and whole cell patch clamping for *I*_h_. In all cases, a colony was selected only if GFP and *I*_h_ was observed in every cell; however, the intensity of GFP expression and *I*_h_ maximal conductance could vary between colonies from the same transfection by a factor of 10, presumably due to differences in the number of copies of plasmid integrated into the host cell genome. Stable cell lines expressing mutant versions of GFP-HCN2 were generated in a similar fashion.

#### Transient Transfections

In our hands, Lipofectamine transfection efficiencies ranged from 5 to 25%. This was not adequate for transient transfection experiments, which required 80–100% transfection efficiency. Therefore, a high efficiency calcium phosphate transfection was employed for immunoprecipitation (IP), biotinylation and patch clamping experiments using transient transfection to increase or decrease SUMOylation. Briefly, cells were plated at ∼60% confluence on 100 or 60 mm cell culture dishes 24 h before transfection. The media was changed at least 1 h before transfection. For a single 100 or 60 mm dish, 25 or 10 μg of total plasmid DNA was diluted in 440 or 250 μl of TE buffer (10 mM Tris-HCl pH8, 1 mM EDTA), respectively. Then, 60 or 30 μl of 2 M CaCl_2_ was added dropwise to the DNA followed by the addition of 500 or 250 μl of 2× HBS (275 mM NaCl, 10 mM KCl, 12 mM Dextrose, 1.4 mM Na_2_HPO_4_, 40 mM HEPES, pH 7.1), respectively. Calcium Phosphate/DNA precipitate was then immediately dropped onto cells. Cells were returned to 37°C and 5% CO_2_ for 4 h followed by either a media change or passaging to 20 mm Poly-L-Lysine coated coverslips and allowed to grow another 24–48 h. Cells were transfected with plasmid DNA expressing either mCherry or a mixture of mCherry + SUMO + Ubc9 or mCherry + SENP1. For co-transfection of multiple plasmids, the total amount of plasmid DNA remained the same (25 or 10 μg) but comprised equal amounts of each of the different plasmids (1:1:1 or 1:1). Transfection efficiency was determined to be the percentage of cells expressing mCherry as judged by fluorescence microscopy. The transfection efficiency of every plate was checked for each experiment, and a plate was only used if the transfection efficiency exceeded 80%.

### Immunoprecipitations

#### GFP-HCN2 IP from Hek Cells

One hundred millimeter plates containing Hek cells were washed twice with ice-cold PBS (137 mM NaCl, 2.7 mM KCl, 10 mM Na_2_HPO_4_, 1.8 mM KH_2_PO_4_, pH 7.4) followed by the addition of RIPA buffer (1% NP40, 50 mM Tris pH 7.4, 150 mM NaCl, 0.1% SDS, 0.5% DOC, 20 mM EDTA, protease inhibitor cocktail at 1:100 and 20 mM NEM) to the plate. Cells were lysed on the plate for 30 min on ice, rocking occasionally. A disposable cell scraper was used to scrape any remaining cell debris from the bottom of the plate, and the lysate was collected and transferred to a sterile tube. Cell debris was pelleted at 12,000 rpm for 10 min at 4°C, and the supernatant was retained. Protein concentration was determined with a BCA assay. IPs were performed using the Classic Magnetic Co-IP Kit (Pierce), following the manufacturer’s instructions. Typically 1 mg of protein was added to the beads and the protein was eluted from the beads in a volume of 100 μl.

#### HCN2 IP from Mouse Forebrain

1.5 mg of the mouse membrane preparation with 5 μg of anti-HCN2 antibody (**Table 1**, Santa Cruz Biotechnology; sc-19708) were used in combination with the Classic Magnetic Co-IP Kit (Pierce), following the manufacturer’s instructions. Protein was eluted from the beads in a volume of 100 μl and 20 μl was used for one lane of an SDS-PAGE gel.

### Western Blotting

Protein samples were run on an SDS-PAGE gel and transferred to a PDVF membrane using a semidry electroblotting system. A membrane was blocked for 1 h in a solution of 5% non-fat powdered milk in TBS (10 mM Tris-HCl pH7.5, 150 mM NaCl), washed for 10 min in TTBS (1x TBS, 0.1% Tween 20), and then incubated overnight at 4°C with the primary antibody, diluted as described in **Table 1**, in 1% non-fat powdered milk in TTBS. The membrane was then washed 3x 5 min each in TTBS and then incubated with the appropriate alkaline phosphatase conjugated secondary antibody, diluted in 1% non-fat powdered milk in TTBS for 2 h at room temperature. Following incubation with the secondary antibody, the membrane was washed 3x 10 min each and Immunstar AP substrate (BioRad) was added to the surface of the membrane, incubated 5 min and then exposed to X-ray film to detect chemiluminescent signals. In some cases, membranes were stripped and reprobed. Stripping involved washing the blot 2x 5 min each in mild stripping buffer [200 mM Glycine, 0.1% SDS, 1% (v/v) Tween 20, pH 2.2]. The blot was then washed 2x 10 min each in PBS and 2x 5 min each in TTBS. The blot was then exposed to film to ensure the signal was removed.

### Patch Clamping Electrophysiology

Coverslips (20 mm) were dipped in ethanol, allowed to air dry, and incubated with Poly-L-Lysine (50 μg/ml in dH_2_O) at 37°C for 1 h. Coverslips were then washed once with sterile dH_2_O, air dried, and stored at 4°C. For an experiment, coverslips were seeded with 8 × 10^4^ cells, and the next day coverslips containing transiently transfected cells were placed into a chamber and constantly perfused with extracellular saline solution (138 mM NaCl, 5.6 mM KCl, 2.6 mM CaCl_2_, 1.2 mM MgCl_2_, 10 mM HEPES, 10 mM Glucose, pH 7.4). Cells were visualized on an Olympus IX70 microscope and transiently transfected cells were identified by fluorescence and only those expressing mCherry were used for whole cell patch clamping experiments. A fire-polished micropipette (Sutter Instruments, 6–8 MΩ) was filled with intracellular solution (10 mM NaCl, 145 mM KCl, 1 mM MgCl_2_, 5 mM HEPES, pH 7.2) and connected to an Axopatch 200B amplifier (Molecular Devices, Foster City, CA, USA). To obtain a whole cell patch, a gentle negative pressure was used to form a ≥1 GΩ seal between the cell membrane and the opening of the micropipette. Suction was used to rupture the cell membrane and recordings were taken from cells that maintained a seal ≥700 MΩ following rupture. Cells were held at -50 mV using pClamp 10 software and *I*_h_ was elicited using 5 s hyperpolarizing voltage steps from -50 to -120 mV in 10 mV increments with 4 s between each step. Steady-state peak current was measured by subtracting the initial fast leak current from the slowly activating *I*_h_ for each hyperpolarizing voltage step. Conductance was calculated using the peak current at each voltage step (*G* = *I*_peak_/(*V*_m_-*V*_rev_); *V*_rev_ *I*_h_ = -35 mV) and fitted to a first order Boltzmann equation.

### Cell Surface Biotinylation

Hek-HCN2 cells plated on 60 mm culture dishes were transiently transfected with either mCherry alone (control) or mCherry + SUMO + Ubc9. Cells were washed twice with ice-cold PBS, supplemented with 0.2 mM CaCl_2_ and 1.5 mM MgCl_2_ (PBS-CM) and incubated for 30 min at 4°C with 2 ml of 0.5–1 mg/ml of EZ-link Sulfo-NHS-SS-Biotin (Thermo Fisher, cat#21331). Cells were washed twice with ice cold PBS-CM, and residual biotin was quenched with PBS-CM plus 100 mM glycine for 15 min at 4°C. Cells were washed once more with PBS-CM followed by lysis with 500 μl of ice cold RIPA buffer (1% NP40, 50 mM Tris pH 7.4, 150 mM NaCl, 0.1% SDS, 0.5% DOC, 2 mM EDTA, protease inhibitor cocktail at 1:100) for 30 min on ice. A disposable cell scraper was used to remove any residual cell debris from the bottom of the plate, and the contents were transferred to a microcentrifuge tube. Cell debris was pelleted by centrifugation at 12,000 rpm for 10 min. The supernatant was removed and immediately incubated for 2 h at 4°C with 100 μl of Neutravidin agarose resin (Pierce, cat #29201) with agitation. The lysate/resin mixture was centrifuged in a spin column (Thermo Fisher, cat #69725) at 1,000 × *g* for 2 min. The flow through containing unbiotinylated intracellular proteins was retained for Western blot (WB) analysis. The resin was washed three times in PBS supplemented with 1% NP40, and 0.1% SDS and three times in PBS supplemented with 0.1% NP40 and 0.5 M NaCl, centrifuging at 500 × *g* for 1 min between each wash. Columns were capped, and biotinylated proteins were eluted from the beads by incubating for 1 h at room temperature with 50 μl of SDS loading buffer (50 mM Tris-HCL pH 6.8, 100 mM DTT, 2% SDS, 0.1% Bromophenol blue, and 10% glycerol). Columns were uncapped and centrifuged at 1,000 × *g* for 2 min to collect the eluted extracellular proteins. Intracellular and extracellular fractions were run on an SDS-PAGE gel and transferred to a PVDF membrane. The membrane was cut at ∼50 kDa. The lower portion of the blot was probed with an anti-actin antibody (Santa Cruz Biotechnology) to confirm that there was no intracellular contamination in the extracellular samples. The top portion of the blot was dually probed with an anti-GFP antibody to detect the GFP-HCN2 channel, and an anti-Na^+^/K^+^-ATPase antibody to use for normalization (**Table 1**). To confirm that expression of the Na^+^/K^+^-ATPase was not varying across treatment groups, samples from control and SUMO+Ubc9 transfected cells were run on a WB. The blot was stained for total protein (SYPRO Ruby blot stain, Bio-Rad) and then probed for the Na^+^/K^+^-ATPase. The Na^+^/K^+^-ATPase signal was normalized by the total protein in the lane. A comparison of the normalized Na^+^/K^+^-ATPase signal across treatment groups indicated that there were no significant differences between control and SUMO+Ubc9 transfected groups (Student’s *t*-test, *p* > 0.05, *n* = 5).

### Image Analysis and Quantification

ImageJ was used to quantify signals from WBs. Optical density (OD) was measured using the Gel Analysis feature of the ImageJ software. Briefly, a rectangular box was drawn around the signal, and ImageJ software was then used to generate a profile plot of the relative intensity. A line was drawn to isolate the area under the curve and set the baseline, thereby subtracting the background, and the area under the curve was measured. In order to ensure images were not saturated, a series of exposures ranging from underexposed to overexposed was always obtained, and only intermediate exposures were quantified. Quantifying the fraction of SUMOylated GFP-HCN2 channels in Hek cells involved measuring a SUMO signal (entire GFP-HCN2 doublet) followed by stripping and reprobing the membrane to obtain a GFP signal (entire GFP-HCN2 doublet). In order to ensure that differences *between* treatment groups were not due to differences created by stripping or variability in the length of SUMO relative to GFP film exposure, a complete set of treatment groups was present on each blot, and measures for each treatment group were obtained from the same exposure/film. However, variability *within* a treatment group could be due to stripping and/or relative lengths of exposures. At best, these experiments are semi-quantitative and the fraction of SUMOylated channels in these cell lines may be over/under-reported with this method. Nevertheless, this method suffices for detecting significant differences between treatment groups.

### Statistical Analysis

All data were analyzed using Prism 7 (Graphpad Software Inc.). Each data set was checked for normality and homogeneity of variance. Data were then analyzed using parametric statistical tests, including Student’s *t*-test and One-way ANOVAs. In all cases, the significance threshold was set at *p* < 0.05. Values that were greater than two standard deviations from the mean were considered statistical outliers and were excluded from the data set. ANOVA’s were followed by *post hoc* tests that either compared all groups to each other (Tukey’s) or to the control (Dunnett’s). Unless otherwise stated all values are presented as the mean ± SEM.

## Results

### Mouse HCN2 Is SUMOylated *In vivo*

**FIGURE 1:**
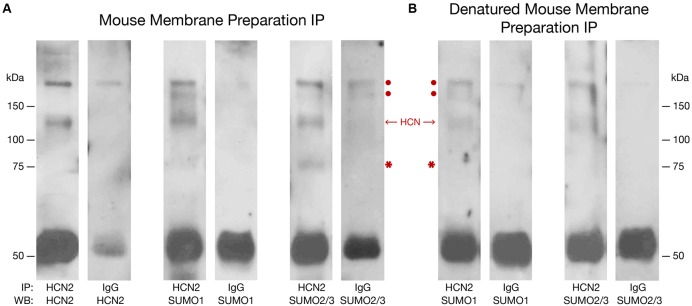
**Mouse HCN2 is SUMOylated *in vivo.*** Mouse forebrain non-denatured **(A)** and denatured **(B)** membrane fractions were used in immunoprecipitation (IP) experiments with an antibody against HCN2 or IgG (negative control). Western blots (WBs) containing the IP products were probed for HCN2, SUMO1, and SUMO2/3. The experiment was repeated three times using the brains from three different mice. Representative WBs are shown from a single experiment. The red dots indicate non-specific products pulled down in the IP. The single band at ∼120 kDa represents HCN2 channels. The asterisk indicates a non-covalently bound, SUMOylated protein. The band at ∼50 kDa represents the IP antibody.

To determine if the mouse HCN2 channel was SUMOylated *in vivo*, an antibody against HCN2 (**Table 1**) or IgG (negative control) was used to IP the channel from mouse forebrain membrane preparations that were or were not denatured, followed by WB analyses using antibodies against HCN2, SUMO1, and SUMO2/3. Note that our anti-SUMO2/3 antibody does not discriminate between SUMO2 vs. SUMO3; therefore, the post-translational modification is referred to as SUMO2/3. **Figure 1** illustrates that HCN2 channels were post-translationally modified by both SUMO1 and SUMO2/3 in the mouse forebrain. Two bands at ∼120 and 75 kDa were observed in the experimental preparations but not in the negative controls. The band at ∼120 kDa was previously identified as the HCN2 channel in mouse membrane preparations (Much et al., 2003). It was observed in blots probed with anti-HCN2, anti-SUMO1, and anti-SUMO2/3 in both non-denaturing and denaturing IP experiments, which suggests both SUMO1 and SUMO2/3 are covalently attached to HCN2 channels in the mouse forebrain. The ∼75 kDa band was detected by the anti-SUMO antibodies, but not by the anti-HCN2 antibody, on the WBs containing IP experiments using non-denatured membrane preparations. Since this ∼75 kDa band was not observed for the denaturing IP experiments, it most likely represents an unknown SUMOylated protein that non-covalently associates with HCN2 channels in the mouse forebrain.

### GFP-HCN2 Channels Are SUMOylated in a Heterologous Expression System

**FIGURE 2:**
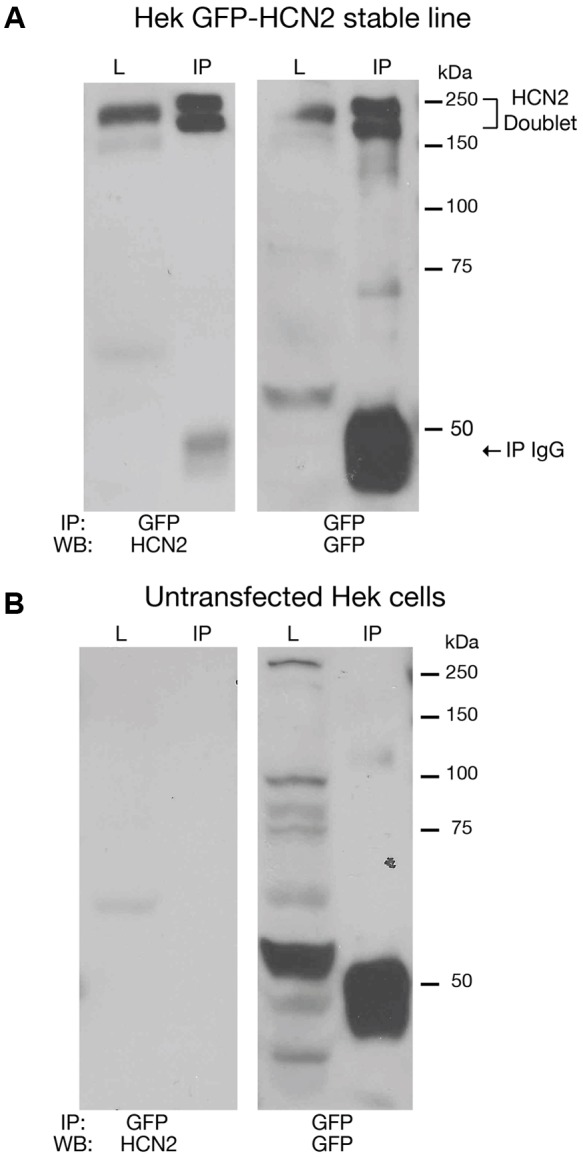
**Establishing a culture system to investigate HCN2 channel SUMOylation.** Cell lysates from a Hek cell line stably expressing GFP-HCN2 channels **(A)** and the parental Hek cell line **(B)** were used in IP experiments with an antibody against GFP. WBs containing the lysate (L) and IP products (IP) were then probed with anti-HCN2 and anti-GFP antibodies. A doublet was recognized by both antibodies in the stably transfected but not parental Hek cell line, suggesting that the doublet represents GFP-HCN2 channels. The band present at 50 kDa in the IP lanes corresponds to the heavy chain of the anti-rabbit antibody used in the IP experiment. The anti-rabbit secondary antibody used in the GFP WB produced a strong signal. The anti-goat secondary antibody used in the HCN2 WB produced a much weaker signal.

Previous work suggested that SUMOylation could be manipulated in tissue culture systems. We therefore generated a Hek-293 cell line stably expressing a GFP-HCN2 fusion protein (Hek-HCN2) as described in “Materials and Methods.” Note that GFP is at the N-terminus of the HCN channel. IP experiments were performed on parental Hek and Hek-HCN2 cell lysates using an anti-GFP antibody followed by WB analyses with anti-GFP or anti-HCN2 antibodies (**Figure 2**). Both the anti-GFP and the anti-HCN2 antibodies detected the same two bands between 150 and 250 kDa (**Figure 2A**). This doublet was not observed in the parental Hek cell line (**Figure 2B**). Together these data suggested that the doublet represented the GFP-HCN2 channel. Previous studies on HCN2 channels expressed in Hek cells showed that the doublet represented two forms of the HCN2 channel (Much et al., 2003; Akhavan et al., 2005; Nazzari et al., 2008). The slower migrating band contained a complex *N*-glycosylation. The faster migrating band instead possessed a simple or no glycosylation. Complex *N*-glycosylation increased the efficiency of surface expression, but channels possessing only a subset of complex *N*-glycosylated subunits (Akhavan et al., 2005) or no complex *N*-glycosylated subunits (Nazzari et al., 2008) could still be detected at the plasma membrane. Relative to past studies, the doublet detected in our experiments is larger, presumably due to the GFP tag.

**FIGURE 3:**
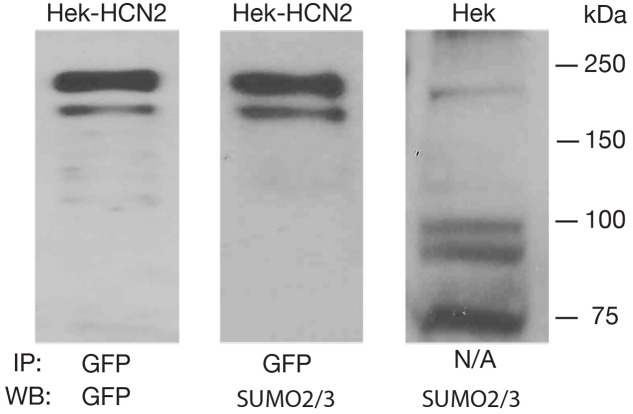
**GFP-HCN2 channels are SUMOylated in Hek-HCN2 cells.** Cell lysates from the Hek-HCN2 cell line were used in IP experiments with an antibody against GFP. WBs containing the IP products were then probed with an anti-GFP antibody or an anti-SUMO2/3 antibody. Parental Hek cell lysates were also probed with the anti-SUMO2/3 antibody. The anti-SUMO2/3 antibody recognized the GFP-HCN2 doublet in the Hek-HCN2 cell line, but not in the parental cell line, suggesting that GFP-HCN2 channels are SUMOylated.

To determine if GFP-HCN2 channels were SUMOylated in our culture system, we performed the same anti-GFP IP with Hek-HCN2 cell lysates, followed by WB analyses using anti-GFP (**Figure 3**, panel 1) and anti-SUMO2/3 (**Figure 3**, panel 2) antibodies. The data indicated that both antibodies recognized the same doublet between 150 and 250 kDa, suggesting that GFP-HCN2 channels were SUMOylated in Hek-HCN2 cells (here termed baseline SUMOylation). The SUMO signal was not due to similarly sized endogenous Hek proteins associating with GFP-HCN2 channels because the anti-SUMO2/3 antibody did not detect the doublet on WBs containing parental Hek cell lysates (**Figure 3**, panel 3).

### Transient Transfection of SUMO2 and Ubc9 Increases HCN2 Channel SUMOylation in a Hek Cell Line Stably Expressing Mouse HCN2

We next wished to manipulate GFP-HCN2 channel SUMOylation in order to study its function. The conjugating enzyme, Ubc9, adds SUMO to a target protein. In some cases, Ubc9 is directed to a SUMOylation site by an external factor, such as a SUMO interaction motif (SIM) domain or an E3 protein (Flotho and Melchior, 2013). In other cases, Ubc9 directly recognizes a consensus sequence and adds SUMO to the K in the consensus sequence. Oftentimes the latter mechanism requires additional stabilizing interactions provided by other proteins or post-translational modifications; however, simply increasing the concentration of the Ubc9 enzyme can obviate their need at some (but not all) consensus sequences, and previous work has demonstrated that SUMOylation of some target proteins can be increased in cell culture by overexpressing SUMO and Ubc9 (Dai et al., 2009). Moreover, SENP has been shown to decrease SUMOylation of some proteins in a heterologous expression system (Dai et al., 2009; Dustrude et al., 2013) despite the fact that SENP has opposing functions: endopeptidase activity removes C-terminal amino acids to activate SUMO and promote target protein SUMOylation; isopeptidase activity removes SUMO conjugated to target proteins to promote deSUMOylation (Yeh, 2009).

**FIGURE 4:**
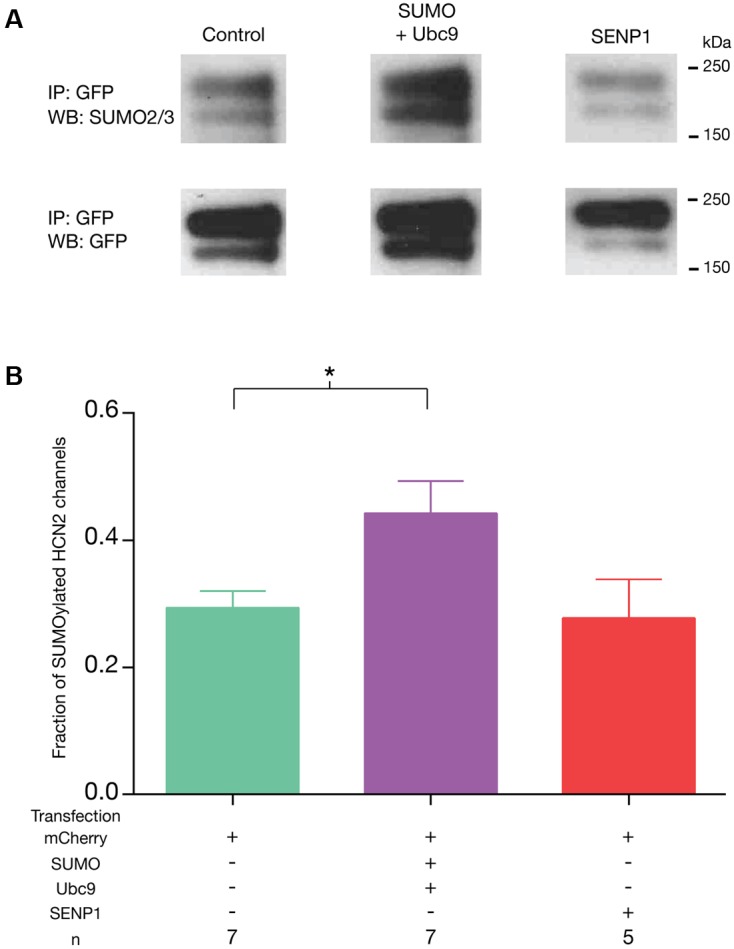
**Transient transfection with SUMO and Ubc9 leads to an increase in GFP-HCN2 channel SUMOylation.** The stable Hek-HCN2 cell line was transiently transfected with mCherry (control), or mCherry + SUMO + Ubc9, or mCherry + SENP1. Two days after transfection, cell lysates were used in IP experiments with an anti-GFP antibody. IP products were resolved with SDS-PAGE and transferred to WBs. Blots were probed with an anti-SUMO2/3 antibody. After recording the result, blots were stripped and reprobed with an anti-GFP antibody. **(A)** Representative blots showing typical chemiluminescent signals for the GFP-HCN2 channel doublet after probing with the anti-SUMO2/3 antibody (upper panel) followed by stripping and re-probing with the anti-GFP antibody (bottom panel). Note that the amount of IP product varied between experiments but not across treatment groups as determined by measures of the GFP OD’s [one-way ANOVA, *F*(2,16) = 0.1285; *p* = 0.8804]. **(B)** The fraction of SUMOylated HCN2 channels in each treatment group (SUMO doublet OD ÷ GFP doublet OD, see text) is plotted as the mean + SEM. The treatment and the *n* are shown below each plot. Each *n* represents a single plate that was transfected and carried through the experiment to produce a single lane on a WB. Asterisk represents a statistically significant difference from control [one-way ANOVA with a Dunnett’s *post hoc*, *F*(2,16) = 4.121; *p* = 0.0360].

In order to investigate whether or not we could manipulate SUMOylation of GFP-HCN2, the Hek-HCN2 cell line was transiently transfected with plasmids expressing mCherry, SUMO2, and Ubc9 or mCherry and SENP1. Control preparations were transfected with mCherry alone. To determine the fraction of HCN2 channels that were SUMOylated in each treatment group, GFP-HCN2 channels were immunoprecipitated from lysates using an anti-GFP antibody. WBs containing the IP products were first probed with an anti-SUMO2/3 antibody. Chemiluminescence was used to detect the SUMO signal, and the OD for the entire GFP-HCN2 doublet was measured with ImageJ (see Materials and Methods). After recording the SUMO signal, the antibody was stripped, and the same blot was reprobed with an anti-GFP antibody. Again, chemiluminescence was used to detect the GFP signal, and the OD of the entire GFP-HCN2 doublet was measured. The fraction of SUMOylated channels was defined as the OD of the SUMO signal divided by the OD of the GFP signal (**Figure 4A**). This non-linear, semi-quantitative method indicated that GFP-HCN2 SUMOylation was significantly increased in the SUMO + Ubc9 group, by an average of 50.7 ± 16.01% relative to the control (**Figure 4B**). There was no significant difference in the amount of SUMOylated channels in cells transfected with SENP1 relative to control. This might suggest a low level of baseline HCN2 SUMOylation in the Hek-HCN2 cell line. On the other hand, since SENP has two opposing functions it is also possible that the balance of these two functions does not change upon SENP1 overexpression, leaving baseline SUMOylation unaltered. In sum, transient transfection of SUMO and Ubc9 increases HCN2 SUMOylation in Hek-HCN2 cells.

### HCN2 Channel SUMOylation Increases *I*_h_ *G*_max_

**FIGURE 5:**
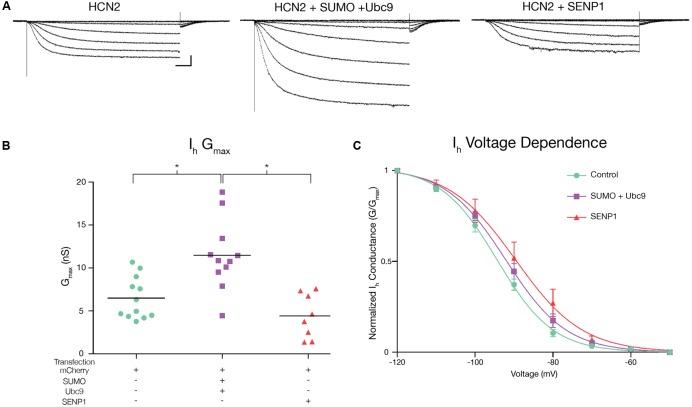
**Increased HCN2 channel SUMOylation augments *I*_h_*G*_max_.** *I*_h_ was measured in Hek-HCN2 cells transiently transfected with either mCherry (control), mCherry + SUMO + Ubc9, or mCherry + SENP1. For each treatment group, data were pooled from ≥3 transfections. **(A)** Representative traces for each treatment group, elicited by stepping the voltage from -50 to -120 mV in 10 mV increments. Scale bars, 500 ms and 200 pA. The kinetics of activation at -120 mV were not altered across treatment groups. Mean activation time constants: control, 400.9 ± 23.01; SUMO + Ubc9, 407.3 ± 33.67; SENP1, 497.4 ± 50.79; one-way ANOVA, *F*(2,32) = 2.224; *p* = 0.1246. **(B)** Plots of *I*_h_ *G*_max_ for each treatment group. Each data point represents a single cell. Bar represents the mean. Transfection with SUMO + Ubc9 significantly increased *I*_h_ *G*_max_ relative to SENP1 and control treatment groups [asterisks, *p* < 0.05; One-way ANOVA with Tukey’s *post hoc*, *F*(2,28) = 13.23; *p* < 0.0001]. **(C)** Plots of voltage dependence of activation. Each data point represents the mean ± SEM. There were no significant differences between treatment groups for mean *V*_50_ [control, -93.88 ± 1.12; SUMO + Ubc9, -91.6 ± 1.29; SENP1, -87.8 ± 3.03; one-way ANOVA, *F*(2,32) = 3.006; *p* = 0.0636] or mean slope [control, -6.61 ± 0.29; SUMO + Ubc9, -7.42 ± 0.41; SENP1, -6.84 ± 0.4; one-way ANOVA, *F*(2,32) = 1.439; *p* = 0.252].

Hyperpolarization of Hek-HCN2 cells to -120 mV consistently elicited an average peak *I*_h_ of 710 pA. This current was never detected in the parental Hek cell line (*n* > 10); thus, it is mediated by GFP-HCN2 channels. We examined if/how SUMOylation affected *I*_h_ using whole cell patch clamp recordings on Hek-HCN2 cell lines that were transiently transfected with mCherry, mCherry + SUMO + Ubc9 or mCherry + SENP1. Transfected cells were identified by mCherry fluorescence. *I*_h_ was elicited by stepping the voltage from -50 to -120 mV in 10 mV increments (**Figure 5A**). The data showed the treatment that produced a ∼50% increase in HCN2 SUMOylation (SUMO + Ubc9, **Figure 4B**) also produced a mean 77 ± 18.8% increase in *I*_h_ *G*_max_ relative to control (**Figure 5B**). Overexpression of SENP1 produced a detectable but not significant mean 31.7 ± 14% decrease relative to control (**Figure 5B**), which was consistent with the finding that SENP1 did not significantly alter baseline SUMOylation of HCN2 channels. The change in *I*_h_ *G*_max_ was not accompanied by any change in the voltage-dependence of activation (**Figure 5C**). Neither the mean V_50_, nor the mean slope of the activation curve were significantly different between control, SUMO + Ubc9, or SENP1 treatment groups (**Figure 5C**). Similarly, the time constant for activation was not significantly different between treatment groups (**Figure 5A**). In sum, increased SUMOylation of GFP-HCN2 produced a corresponding increase in *I*_h_ *G*_max_ with no change in voltage dependence or kinetics of activation.

### SUMOylation Increases HCN2 Channel Surface Expression

**FIGURE 6:**
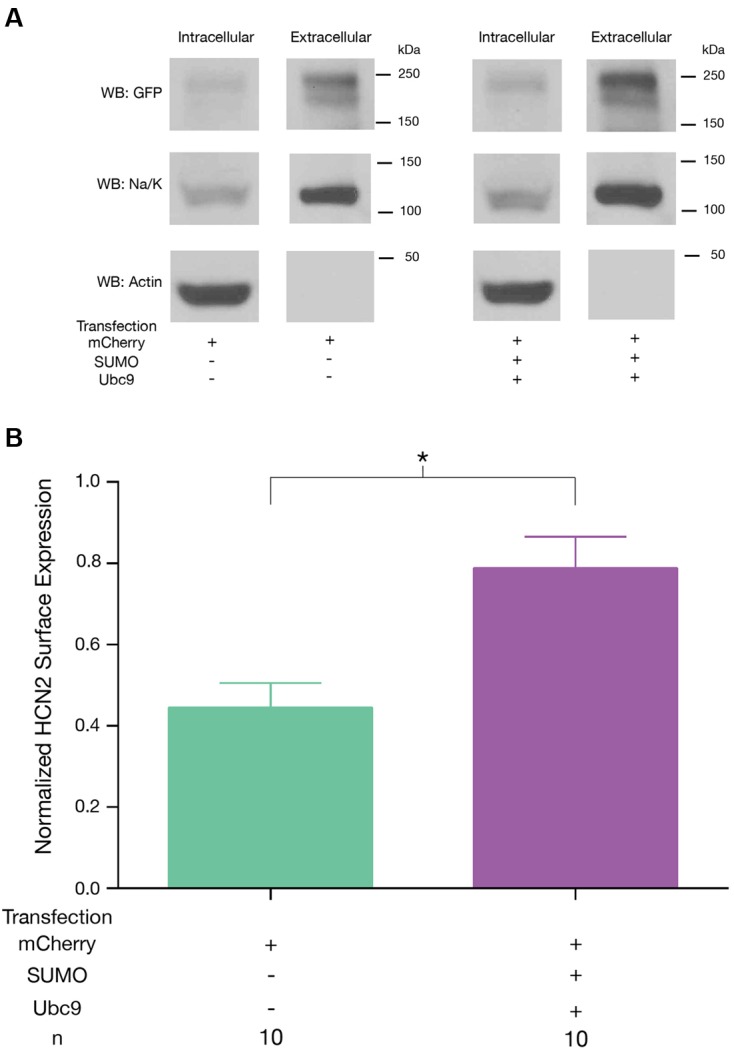
**Increased SUMOylation augments HCN2 channel surface expression.** GFP-HCN2 channel cell surface expression was monitored using a biotinylation assay. Hek-HCN2 cells were transiently transfected with mCherry alone or mCherry + SUMO + Ubc9. Two days after transfection cultures were biotinylated and cell surface proteins were isolated from cell lysates using Neutravidin. Both the intracellular and cell surface fractions were run on a WB and probed with antibodies recognizing GFP, Na^+^/K^+^-ATPase, and Actin. **(A)** Representative WBs. **(B)** Plots depicting average normalized GFP-HCN2 channel surface expression (GFP doublet OD ÷ Na^+^/K^+^-ATPase OD). The treatment and the *n* are shown below the graph. Each *n* represents a single plate that was transfected, biotinylated and carried through the experiment to produce a single lane on a WB. Asterisk indicates significant difference between treatment groups (Student’s *t*-test, *p* < 0.05).

We next tested whether or not the SUMOylation-dependent increase in *I*_h_ *G*_max_ was accompanied by an increase in GFP-HCN2 channel surface expression. A biotinylation assay was used on Hek-HCN2 cells that were transiently transfected with mCherry or mCherry + SUMO + Ubc9. After 2 days, transfected cells were incubated with biotin, which binds to cell surface proteins. The cells were then lysed, and the biotinylated proteins were pulled out using Neutravidin. The unbiotinylated (intracellular) and biotinylated (extracellular surface) proteins were run on an SDS-PAGE gel and transferred to a WB, which was subsequently divided at ∼50 kDa. The portion of the blot containing proteins <50 kDa was probed with an antibody against actin. The ∼37 kDa band corresponding to actin was only seen in the intracellular fractions, indicating there were no intracellular proteins contaminating the extracellular surface samples (**Figure 6A**). The section of the blot corresponding to proteins >50 kDa was dually probed with an anti-GFP antibody and an antibody against the Na^+^/K^+^-ATPase (**Figure 6A**). The doublet was detected with the anti-GFP antibody. The band at ∼120 kDa represents the Na^+^/K^+^-ATPase. The OD of the GFP signal (entire GFP-HCN2 doublet) was normalized by that for the Na^+^/K^+^-ATPase signal. We observed a significant mean 70.6 ± 18.2% increase in HCN2 surface expression in cells transfected with SUMO + Ubc9 relative to control (**Figure 6B**). Based on these findings, we concluded that the ∼77% increase in *I*_h_ *G*_max_ observed upon enhanced GFP-HCN2 channel SUMOylation was due, at least in part, to a ∼70% increase in the surface expression of the GFP-HCN2 channel.

### Only One of Six Putative SUMOylation Sites Is Necessary for the Increase in GFP-HCN2 Surface Expression and *I*_h_ *G*_max_ Elicited by Overexpression of SUMO and Ubc9

**FIGURE 7:**
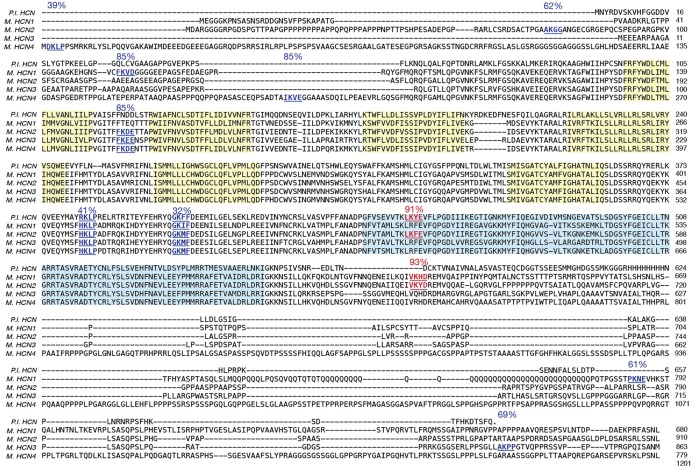
**Identification of HCN2 channels SUMOylation sites.** Amino acid sequences for the vertebrate and invertebrate HCN channels were aligned: mouse HCN1 (NP_034538), mouse HCN2 (NP_032252), mouse HCN3 (NP_032253), mouse HCN4 (NP_001074661), spiny lobster HCN (ABI94038). Putative SUMOylation sites predicted by SUMOplot freeware are indicated in blue (not mutated this study) or red (mutated in this study). The six transmembrane domains (yellow) and CNBD (blue) have been highlighted.

GFP-HCN2 channels can be SUMOylated in our culture system, and increased SUMOylation leads to an augmentation of *I*_h_ *G*_max_ due, at least in part, to enhanced GFP-HCN2 channel surface expression. Approximately 63% of all SUMOylation occurs within a SUMOylation consensus sequence (Hendriks et al., 2015), e.g., ΨKXD/E, with Ψ being a hydrophobic residue and X being any amino acid (Rodriguez et al., 2001; Sampson et al., 2001; Flotho and Melchior, 2013). We next investigated if HCN channels possessed SUMOylation consensus sequences. SUMOplot freeware^1^ was used to identify potential HCN SUMOylation sites for all mouse HCN channel isoforms and for the spiny lobster HCN channel. The amino acid sequences for all five channels were aligned, SUMOylation consensus sequences were highlighted for all isoforms and the probability of SUMOylation, as calculated by SUMOplot, was indicated (**Figure 7**). There were six putative HCN2 SUMOylation sites with probabilities ranging from 32 to 93%. One site was extracellular (K210) and was not considered further. Two of the five intracellular HCN2 consensus sequences (K464 and K484) were observed in all mammalian HCN channel isoforms and in the lobster HCN channel. The other three consensus sequences were isoform/species specific. The consensus sequence containing K669 was exclusive to mouse HCN1 and HCN2 channels. The consensus sequence that included K534 was found only in mouse HCN2 and lobster HCN channels. The consensus sequence containing K75 was unique to the mouse HCN2 channel. Additional putative SUMOylation sites not observed in mouse HCN2 channels existed in other mouse and lobster HCN channel isoforms.

**FIGURE 8:**
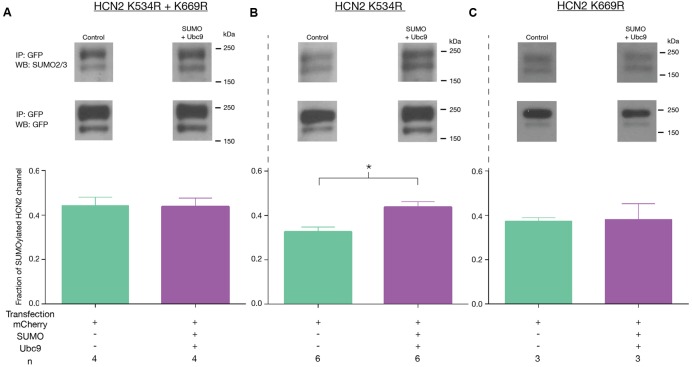
**Overexpression of SUMO and Ubc9 enhances SUMOylation at K669.** The three mutant cell lines **(A–C)** were transiently transfected with mCherry or mCherry + SUMO + Ubc9. Cell lysates were used in IP experiments with anti-GFP antibodies. WBs containing IP products were probed with the anti-SUMO2/3 antibody, stripped and reprobed with the anti-GFP antibody. *Top Panel*: Representative blots showing the GFP-HCN2 doublet that was measured. *Bottom Panel:* Plots of the fraction of SUMOylated GFP-HCN2 channels in each mutant cell line, mean + SEM. The treatment and the *n* are shown below the graph. Each *n* represents one plate that was transfected and carried through the experiment to produce a single lane on a WB. Asterisk significantly different (Student’s *t*-test, *p* < 0.05).

We next investigated if HCN2 consensus sequences were substrates for SUMOylation in our culture system. Because K534 and K669 showed a greater than 90% probability of SUMOylation (**Figure 7**), we chose to examine these first. These two sites were mutated individually and together by changing the positively charged K residue to a positively charged arginine (R), which should prevent SUMO conjugation without disrupting charge interactions (Feliciangeli et al., 2007). Three Hek cell lines stably expressing the mutated channels were generated: Hek-HCN2 K534R + K669R, Hek-HCN2 K534R, and Hek-HCN2 K669R. Each stable mutant cell line was transiently transfected with mCherry or mCherry + SUMO + Ubc9. After ∼48 h the fraction of SUMOylated GFP-HCN2 channels was measured as previously described for **Figure 4**. Based on our semi-quantitative measures, the mutations did not appear to alter baseline HCN2-GFP SUMOylation in the mCherry treatment group (mean ± SEM; wild-type = 0.31 ± 0.025, K534R + K669R = 0.41 ± 0.04, K534R = 0.31 ± 0.02, and K669R = 0.37 ± 0.015; One-way ANOVA *F*(3,19) = 2.644, *p* = 0.0788). On the other hand, the mutations did disrupt the increase in HCN2-GFP SUMOylation normally elicited by SUMO + Ubc9 overexpression (**Figure 8**). Increased SUMOylation was no longer observed in the Hek-HCN2 K534R + K669R cell line (**Figure 8A**) or the Hek-HCN2 K669R cell line (**Figure 8C**); however, the Hek-HCN2 K534R cell line still displayed increased SUMOylation upon SUMO + Ubc9 overexpression (**Figure 8B**). In sum, the data suggest that overexpression of SUMO + Ubc9 increases SUMOylation at K669; furthermore, because SUMOylation is still observed when this site is mutated, at least one additional site on the GFP-HCN2 channel must be SUMOylated.

**FIGURE 9:**
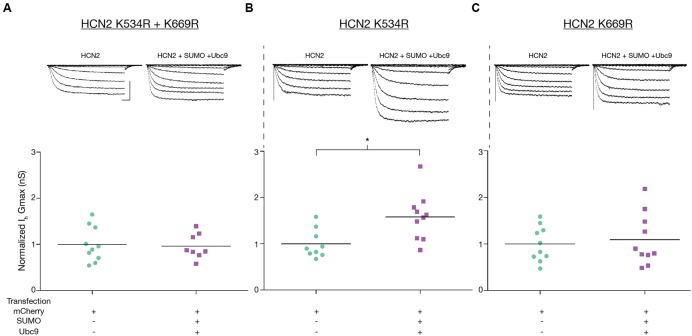
**Enhanced SUMOylation at K669 augments *I*_h_*G*_max_.** *I*_h_ *G*_max_ was measured following transient transfection with mCherry or mCherry + SUMO + Ubc9 in the three mutant cell lines **(A–C)**. *Top panel*: Representative traces for each treatment group for each mutant cell line. Scale bars, 500 ms, and 500 pA, *Bottom Panel*: Plots of normalized *I*_h_ *G*_max_ (*G*_max_ ÷ mean mCherry *G*_max_) for each mutant cell line. *I*_h_ *G*_max_ was normalized to account for differences in overall expression of the HCN2 channels between the different mutant cell lines, most likely due to difference in the copy number of the plasmid integrated into the genome. Each data point represents a single cell. Asterisk significantly different (Student’s *t*-test *p* < 0.05, recordings were pooled from ≥3 separate transfections).

We next examined *I*_h_ *G*_max_ in the mutant cell lines. Consistent with the previous finding, we observed that mutating K669 but not K534 prevented the increase in *I*_h_ *G*_max_ upon SUMO + Ubc9 overexpression. *I*_h_ *G*_max_ was not significantly different between the SUMO + Ubc9 vs. control treatment groups in Hek-HCN2 K534R + K669R (**Figure 9A**) or Hek-HCN2 K669R cell lines (**Figure 9C**). However, in Hek-HCN2 K534R cells, a 58.2 ± 16.1% increase in *I*_h_ *G*_max_ was still observed in the SUMO + Ubc9 treatment group relative to control (**Figure 9B**). Together these data suggest that SUMOylation of K669 is responsible for the increase in *I*_h_ *G*_max_ observed upon overexpression of SUMO+Ubc9.

**FIGURE 10:**
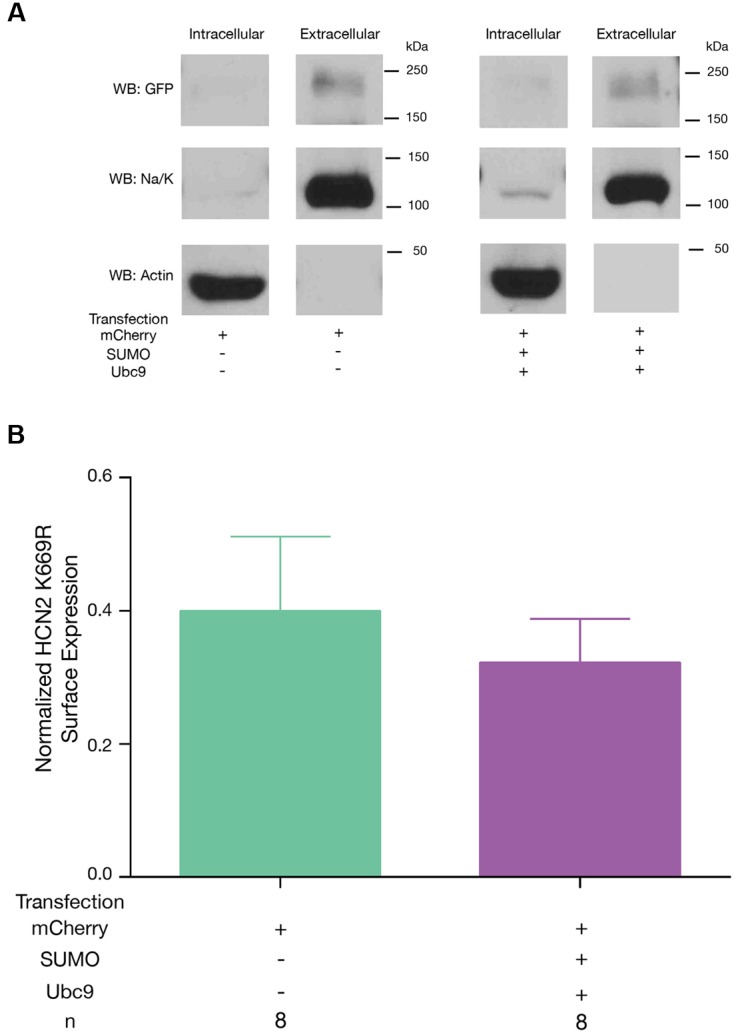
**Enhanced SUMOylation at K669 increases GFP-HCN2 channel surface expression.** The K669R mutant cell line was transiently transfected with mCherry or mCherry + SUMO + Ubc9, followed by the biotinylation assay to measure GFP-HCN2 surface expression. **(A)** Representative blots probed with anti-GFP, anti-Na/K ATPase, and anti-actin antibodies. **(B)** Plots of normalized GFP-HCN2 channels in each treatment group. The treatment and the *n* are shown below the graph. Each *n* represents a single plate that was transfected, biotinylated, and carried through the experiment to produce a single lane on a WB. There was no significant difference between treatment groups (Student’s *t*-test, *p* = 0.5606).

Lastly, we tested if mutating K669 prevented the increase in GFP-HCN2 surface expression normally elicited by overexpression of SUMO and Ubc9. Hek-HCN2 K669R cells were transiently transfected with mCherry or mCherry + SUMO + Ubc9 and the biotinylation assay was performed as described for **Figure 6**. The data indicated then when K669 was mutated, overexpression of SUMO and Ubc9 could no longer elicit an increase in GFP-HCN2 surface expression (**Figure 10**). In sum, the K669R mutation blocked the increase in SUMOylation, *I*_h_ *G*_max_ and surface expression normally elicited by overexpression of SUMO + Ubc9. This suggests that enhanced SUMOylation at K669 augments GFP-HCN2 surface expression which in turn amplifies *I*_h_ *G*_max_.

## Discussion

Ion channels are emerging as targets of SUMOylation. The extent to which different classes of ion channels are SUMOylated and the function and regulation of ion channel SUMOylation are only beginning to be investigated. Here we report, for the first time, that HCN channels are SUMOylated *in vivo* and in a heterologous expression system. IP experiments with mouse forebrain membrane preparations followed by Western blotting showed that mouse HCN2 channels were post-translationally modified by SUMO1, as well as SUMO2 and/or SUMO3 *in vivo*. HCN2 channels were also SUMOylated under baseline conditions in a Hek cell line stably expressing a GFP-HCN2 construct, and transient transfection with SUMO and the SUMO-conjugating enzyme, Ubc9, increased GFP-HCN2 channel SUMOylation above baseline. This, in turn, augmented GFP-HCN2 channel surface expression and produced a corresponding increase in *I*_h_ *G*_max_ without altering the voltage dependence or kinetics of activation. Using site-directed mutagenesis of HCN2 and Hek cell lines stably expressing mutant GFP-HCN2 channels, we showed that SUMOylation at amino acid K669 was increased upon overexpression of SUMO + Ubc9, and this alone was responsible for enhanced GFP-HCN2 channel surface expression and augmentation of *I*_h_ *G*_max_. However, the K669R mutation, which prevented SUMOylation at this site, did not alter baseline SUMOylation of HCN2 channels, suggesting an additional site(s) on the GFP-HCN2 channel may be SUMOylated under baseline conditions.

### Ion Channels Are SUMOylated *In vivo*

The effect of SUMO modification on ion channels has become an area of increasing interest. Previous studies have identified additional ion channel targets of SUMOylation, and the effect of SUMOylation on these channels is quite diverse. For example, SUMO modification of Kv2.1 inhibits the current by speeding time-dependent inactivation and slowing recovery from inactivation in pancreatic cells (Dai et al., 2009). However, SUMOylation of the same channel in hippocampal neurons results in a depolarized shift in the voltage dependence of activation, making the neurons more excitable (Plant et al., 2011). Studies looking at SUMOylation of the Kv1.5 channel found that it produced a shift in the voltage dependence of inactivation (Benson et al., 2007). A recent study showed that in SENP2 null mice hyperSUMOylation of Kv7 channels diminished the M-current and resulted in neuronal hyperexcitability, leading to seizures and sudden death (Qi et al., 2014). In addition to altering the biophysical properties of ion channels, SUMOylation can alter their surface expression. For example, kainite receptor SUMOylation leads to internalization of the receptor, and this effect can be enhanced by agonist binding and PKC phosphorylation (Martin et al., 2007; Konopacki et al., 2011; Chamberlain et al., 2012). Here, we have shown that HCN2 channel SUMOylation at K669 increases surface expression and *I*_h_ *G*_max_. It is important to note that the change in surface expression may not be the sole explanation for the change in *I*_h_ *G*_max_; though it was not examined here, changes in biophysical properties, such as an increase in the open probability (Po) of the channel, could also contribute to the observed increase in *I*_h_ *G*_max_. In addition, silent channels in the membrane could be activated by enhancing protein–protein interactions.

### HCN Channels Contain Multiple SUMOylation Consensus Sequences

The conjugating enzyme, Ubc9, adds SUMO to target proteins. In some cases, Ubc9 is directed to a SUMOylation site by an external factor, such as a SIM domain or an E3 ligase. In these instances, SUMOylation does not necessarily occur at a known consensus sequence (Flotho and Melchior, 2013). In other cases Ubc9 itself recognizes a consensus sequence and adds SUMO to the K in the consensus sequence. One study indicates that SUMOylation at known consensus sequences accounts for ∼63% of all SUMOylation, although this may vary with the condition, e.g., normal growth conditions vs. heat shock (Hendriks et al., 2015). There are five predicted intracellular SUMOylation consensus sequences in HCN2 (**Figure 7**). Using Hek cell lines stably expressing wild type and mutant GFP-HCN2 channels, we clearly demonstrated that overexpression of SUMO and Ubc9 increased SUMOylation at only one of the five sites, K669. However, when K669 was mutated, GFP-HCN2 channel SUMOylation was still observed, suggesting additional sites were SUMOylated under baseline conditions. Since additional consensus sequences exist on both the GFP tag and the HCN2 channel, either or both could contribute to the observed baseline SUMOylation. It is not clear why SUMOylation did not increase at these site(s) upon overexpression of SUMO and Ubc9. They may be maximally SUMOylated and/or additional proteins or regulatory mechanisms may be necessary for SUMOylation to occur. Alternatively, baseline SUMOylation may not occur at consensus sequences, in which case increasing SUMO and Ubc9 should have no effect.

The five putative HCN2 consensus sequences ranged from being unique to HCN2 channels to being conserved across all mouse HCN and lobster channel isoforms. The consensus sequence that is known to be SUMOylated and which contains K669 is present exclusively in mouse HCN1 and HCN2 channels. Our data indicated that SUMOylation at K669 was not necessary to produce a functional current: *I*_h_ was not observed in whole cell patch clamp recordings from parental Hek cells, but it was recorded in Hek cells stably expressing GFP-HCN2 channels with a K669R mutation that prevented SUMOylation at this site. Although K669 SUMOylation was not necessary, increased SUMOylation at K669 augmented GFP-HCN2 channel surface expression and *I*_h_ *G*_max_. These data suggest that SUMOylation at K669 may alter the rates of channel trafficking to and/or from the plasma membrane and/or alter channel retention in the plasma membrane. The surface expression of HCN1 channels can be rapidly adjusted in response to changes in activity, including seizures (Shah et al., 2004; Noam et al., 2010; Jung et al., 2011). Activity can also regulate the SUMOylation of target proteins and the expression and localization of SUMO and the SUMOylation machinery in neurons (Jaafari et al., 2013; Loriol et al., 2013; Sun et al., 2014). Thus, it is possible that changes in activity might alter HCN channel surface expression by altering SUMOylation at this conserved site.

The putative HCN2 channel SUMOylation sites at K464 and K484 were observed in all mouse HCN channel isoforms and even in the lobster HCN channel (**Figure 7**), suggesting that they may play important functional roles. Both K464 and K484 are in the C-linker that connects the CNBD to the transmembrane region. The C-linker is necessary for channel trafficking and cAMP gating (Ulens and Siegelbaum, 2003; Zagotta et al., 2003; Zhou et al., 2004). The C-linker contains seven α-helices, A–F (Zagotta et al., 2003); K464 lies between α-helices A and B while K484 lies between α-helices B and C. Since SUMOylation is thought to occur only in regions lacking secondary structure, it is noteworthy that both putative SUMOylation sites lie just outside of the α-helices. The A and B α-helices in one subunit are thought to interact with the C and D α-helices in a neighboring subunit (Zagotta et al., 2003). We have not yet tested whether these two SUMOylation sites are involved in channel folding, assembly and/or trafficking, but it may be that SUMOylation plays a fundamental role in these processes for all HCN channels.

The K534 SUMOylation consensus site lies within the CNBD, which is also necessary for trafficking and cAMP gating (Akhavan et al., 2005). According to a previous crystallography study of the solubilized C-terminus (Zagotta et al., 2003), this site lies within α-helix A of the CNBD. The K534R mutation did not appear to alter baseline SUMOylation in our studies, nor did it prevent the increase in HCN2 SUMOylation observed upon overexpression of SUMO + Ubc9. Together these data suggest that K534 may not be SUMOylated in the mature channel. However, this site was assigned a high score by SUMOplot, and it is conserved across species, which often suggests functionality. Since SUMOylation is a dynamic and highly regulated process (Watts, 2013), it may be that this site is transiently SUMOylated in response to a specific signal not mimicked in our experiments.

### Potential SUMO-Dependent HCN2 Channel Interactions

SUMOylation can produce several distinct physiological consequences, but its primary function is to facilitate protein–protein interactions through the binding of a SUMO post-translational modification on the target protein to a SIM on the interacting partner protein (Hickey et al., 2012). Other interaction motifs also exist (Flotho and Melchior, 2013). We have demonstrated that HCN2 channel SUMOylation can regulate surface expression, and that (putative) HCN2 SUMOylation sites are located in domains that are important for channel trafficking and surface expression (Proenza et al., 2002; Tran et al., 2002; Akhavan et al., 2005; Nazzari et al., 2008). Thus, while SIM containing interaction partners for HCN2 channels have not yet been identified, it seems likely that they may include trafficking and scaffold proteins. HCN2 synthesis and assembly begin in the ER where heavily glycosylated subunits associate through N- and C-terminal domains (Proenza et al., 2002; Tran et al., 2002). Channels transit from the ER, through the Golgi and to the plasma membrane via their interactions with a host of proteins that mediate vesicular trafficking. Plasma membrane HCN2 channels can be recycled. Recycled HCN channels are stored in the endosome recycling compartments below the plasma membrane until a signal, such as Phospholipase D activation, triggers insertion back into the plasma membrane (Hardel et al., 2008). SUMOylation could be involved at any of these trafficking stages. Scaffold and trafficking proteins are known to interact with three distinct domains in the C-terminus of the HCN2 channel: the C-linker, the CNBD, and the ∼230 amino acids distal to the CNBD (Kimura et al., 2004; Santoro et al., 2004; Lewis et al., 2009; Han et al., 2011). S-SCAM, mint, and talamin are scaffold and trafficking proteins that form an assemblage with HCN2 channels through unknown interactions involving the distal ∼230 amino acids. SUMOylation site K669 is located in this distal fragment, suggesting that SUMOylation at this site may enhance surface expression by promoting these assemblages (Han et al., 2011). SUMOylation in the C-linker and/or the distal fragment could also potentially promote or prevent the binding of trafficking/scaffolding proteins like TRIP8b (Santoro et al., 2004; Lewis et al., 2009; Han et al., 2011) and S-SCAM (Kimura et al., 2004) to the CNBD.

### HCN Channel SUMOylation and Neurological Disorders

Multiple neurological disease states may be linked to aberrant SUMOylation. Normally SUMOylation of α-synuclein promotes a functional, soluble conformation of the protein, but deSUMOylation leads to α-synuclein aggregation and cytotoxicity, which are hallmarks of Parkinson’s disease (Eckermann, 2013). Also, the parkin gene is frequently mutated in autosomal recessive Parkinson’s patients (Guerra de Souza et al., 2016). Parkin is a ubiquitin ligase that mediates the degradation of misfolded mitochondrial proteins. SUMOylation regulates parkin shuttling from the cytosol to the nucleus, thereby controlling its availability at the mitochondria (Um and Chung, 2006). Alzheimer’s disease is characterized by the formation of neurofibrillary tangles and amyloid-beta (Aβ)-containing plaques. Two proteins closely associated with these features, amyloid precursor protein (APP) and tau, have recently been identified as targets of SUMOylation (Hoppe et al., 2015). Their improper SUMOylation could contribute to misfolding and aggregation (Flotho and Melchior, 2013). Altering the expression of enzymes in the SUMOylation pathway can lead to seizures due, at least in part, to hyperSUMOylation of Kv7 channels (Qi et al., 2014). If SUMOylation is globally disrupted in a given disease, then multiple targets could be affected, including HCN2 channels. Indeed, HCN2 channel activity is progressively reduced in a mouse model of Parkinson’s disease leading to altered pacemaking in globus pallidus neurons (Chan et al., 2011), and altered HCN2 channel expression has been associated with seizures in a variety of instances (Ludwig et al., 2003; DiFrancesco et al., 2011; Nakamura et al., 2013; DiFrancesco and DiFrancesco, 2015). Future studies on the function and regulation of HCN channel SUMOylation could provide important insights into HCN2 channel dysfunction in these disease states.

## Ethics Statement

All animal procedures were conducted in compliance with the regulation of the Institutional Animal Care and Use Committee of Georgia State University.

## Author Contributions

Authors AP and DB were responsible for the conception and design of the research presented here. Authors AP, MW, LF, ST, JD, and DB provided substantial contribution to the acquisition and analysis of the data presented here. All authors also provided input during the drafting and revision of the manuscript.

## Conflict of Interest Statement

The authors declare that the research was conducted in the absence of any commercial or financial relationships that could be construed as a potential conflict of interest.

## Footnotes

1 www.abgent.com/sumoplot
